# Acute arterial thrombosis during adjuvant Adriamycin-cyclophosphamide chemotherapy in a patient with early breast cancer: A case report

**DOI:** 10.1097/MD.0000000000018249

**Published:** 2019-12-16

**Authors:** Lee Chan Jang, Young Jin Choi

**Affiliations:** Department of Surgery, College of Medicine, Chungbuk National University, Cheongju, Republic of Korea.

**Keywords:** Adriamycin-cyclophosphamide, arterial thrombosis, breast carcinoma, chemotherapy, thrombectomy

## Abstract

**Rationale:**

Cancer and chemotherapy individually confer hypercoagulability and increased risks of thrombosis. Most thromboembolic complication after breast cancer chemotherapy was venous thrombosis after multiagent chemotherapy. Arterial thrombosis is extremely rare in early breast cancer patients receiving adjuvant chemotherapy.

**Presenting concerns:**

A 55-year-old woman with right breast cancer presented to the emergency department with sudden pain, numbness, and swelling in her left hand. She underwent breast conserving surgery and sentinel lymph node biopsy 2 months before the visit. She received the second cycle of adjuvant Adriamycin-cyclophosphamide chemotherapy 5 days before.

**Interventions:**

Computed tomography angiography revealed acute arterial thrombosis in the left brachial, radial, and ulnar arteries. Unfractionated heparin was initiated immediately, followed by brachial and radial-ulnar thrombectomy, restoring perfusion to the extremity. The postoperative course was uncomplicated; she was discharged on warfarin at a daily dose of 4 mg.

**Outcomes:**

Chemotherapy was discontinued. Anticoagulation with warfarin was continued. She subsequently received adjuvant endocrine therapy with an aromatase inhibitor and adjuvant radiotherapy.

**Main lessons:**

Despite the low risks of arterial thrombosis in breast cancer, it is a devastating complication with significant morbidity and mortality. Thromboprophylaxis should be considered in those at risk. Immediate anticoagulant therapy and surgical intervention should be considered in affected cases.

## 1 Introduction

Cancer is associated with an increased incidence of thrombosis. Up to 15% of patients with clinically overt cancer present with venous thromboembolism (VTE) during the course of their disease.^[1]^ This prothrombotic state may be attributed to the ability of tumor cells to directly activate the coagulation cascade; this causes thrombosis or induces procoagulant properties, and inhibits the anticoagulant properties of vascular endothelial cells, platelets, monocytes, and macrophages.^[2]^

The prothrombotic tendency may be further enhanced by anticancer treatments such as surgery, chemotherapy, and various other antineoplastic and supportive therapies. Surgery is a well-known precipitating factor for thromboembolic disease as the hemostatic system is activated in the perioperative period. Anticancer drugs may also cause thrombosis. The annual incidence of VTE in patients receiving chemotherapy is estimated to be 11%.^[3]^ For many years, various chemotherapeutic agents such as 5-asparaginase, cisplatin, thalidomide, mitomycin-C, and fluorouracil have been associated with thromboembolic complications.^[4]^ The mechanisms include release of procoagulants and cytokines by tumor cells, damage to the vascular endothelium, and stimulation of tissue factor activity in monocytes and macrophages.^[1]^ Antihormonal therapies including tamoxifen, targeted agents including bevacizumab, and many supportive agents including hematopoietic growth factors and corticosteroids are associated with an increased risk of thrombosis.^[4]^

Breast cancer is associated with a low incidence of thromboembolic events (TEE) compared to other cancers. The risk of deep vein thrombosis (DVT) in patients with early stage breast cancer receiving chemotherapy is 2% to 10%, compared to less than 1% in those not receiving it.^[5]^ Conventional combination chemotherapy regimens including CMFVP (cyclophosphamide, methotrexate, fluorouracil, vincristine, and prednisolone) and CMF (cyclophosphamide, methotrexate, and fluorouracil) are known to increase the risk of thromboembolism.^[6,7]^ The phlebographically proven incidence of DVT with drugs used in contemporary practice, such as epirubicin and cyclophosphamide, is 10%.^[8]^ Following multiagent chemotherapy for breast cancer, the most common thromboembolic complication is venous thrombosis; arterial thrombosis is an extremely rare event, with a reported incidence of 1.0% to 4.8%.^[9–11]^ Despite the low risk of arterial thrombosis in patients with breast cancer, it is a potentially devastating complication that results in significant morbidity and mortality.

In the present report, we describe an extremely rare case of acute arterial thrombosis in the upper extremity in a patient receiving adjuvant chemotherapy with Adriamycin-cyclophosphamide for completely resected stage I breast cancer. The publication of this report was approved by the Institutional Review Board of the Chungbuk National University Hospital, Republic of Korea. The patient had provided informed consent for her treatment and had agreed to the publication of the figures and data in this report.

## 2 Presenting concerns

**Figure 1:**
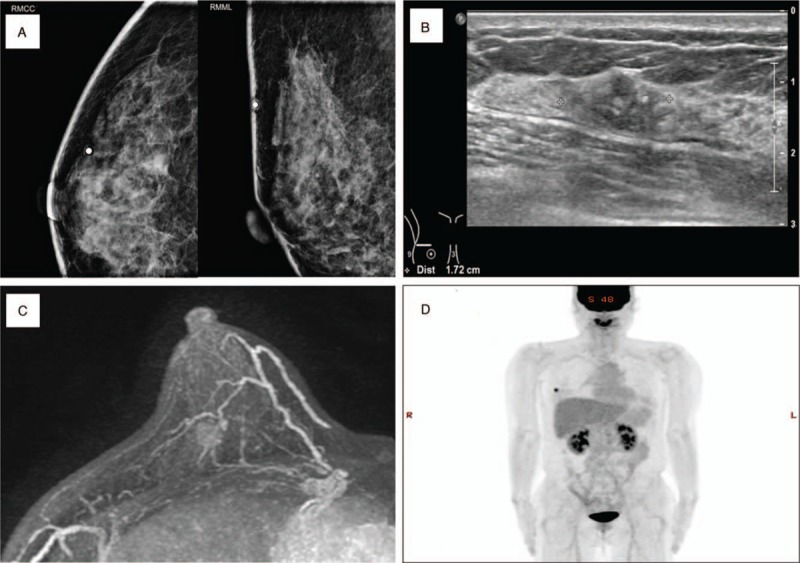
Mammography (A), breast ultrasonography (B), breast magnetic resonance imaging (MRI) (C) and positron emission tomography-computed tomography (PET-CT) (D) findings showing irregular enhancing mass in the right breast without axillary lymph node metastasis. There was no evidence of distant metastasis.

A 55-year-old postmenopausal woman presented to the emergency department with sudden pain, numbness, and swelling in her left hand. She had been diagnosed invasive ductal carcinoma of the right breast 2 months before the visit. The radiologic tests, which included computed tomography (CT) scans of the chest, abdomen, and pelvis, and a positron emission tomography computed tomography (PET CT), showed no evidence of distant metastatic disease (Fig. 1). She underwent right sided breast conserving surgery and sentinel lymph node biopsy. The tumor was found to be moderately differentiated invasive ductal carcinoma, measuring 1.5 cm in diameter. On immunohistochemistry, the tumor tested positive for the estrogen receptor (2+, 70%) and progesterone receptor (1+, 10%), and negative for C-erb-B2; the Ki 67 proliferation index was high at 80%. The 3 sentinel lymph nodes sampled were negative for malignancy. The surgical margins were negative; according to the 8th edition of the American Joint Committee on Cancer (AJCC) staging system, the tumor was of pathologic stage I (pT1cN0M0).

## 3 Clinical findings

The patient had a strong family history of breast and ovarian cancer. Her older sister was diagnosed with breast cancer at 46 years of age and her 2 younger sisters were diagnosed with breast and ovarian cancer at the ages of 53 and 51 years, respectively. Tests for mutations in the BRCA1 and BRCA2 genes had shown a variant of uncertain significance in BRCA1, with no abnormality of BRCA2.

She was within the normal range for body weight, with a body mass index (BMI) of 22.9 kg/m^2^. She had been diagnosed with hypertension 15 years before and had never undergone hormone replacement therapy in the past. She was a never-smoker and denied any other significant medical history including that of diabetes, atherosclerotic disease, or tuberculosis. She was only taking Amlodipine (10 mg) daily. The pre-treatment laboratory studies, which included a complete blood count and a screening complete metabolic panel, were within normal limits. The baseline prothrombin time (PT), prothrombin time/international normalized ratio (PT/INR), and activated partial thromboplastin time (aPTT) were 11.8 seconds (10.1–13.1 seconds), 1.00 (<1.2), and 25.0 seconds (24.0–33.0 seconds), respectively. The baseline cardiac evaluation, which included an electrocardiogram and echocardiogram, were within normal limits. Her family and personal history were not indicative of either thromboses or bleeding disorders.

On examination, her Eastern Cooperative Oncology Group (ECOG) performance status score was 1. Chemotherapy, consisting of Adriamycin (60 mg/m^2^) and cyclophosphamide (600 mg/m^2^) was initiated 18 days after surgery. The patient was scheduled to receive 4 cycles of Adriamycin-cyclophosphamide chemotherapy every 3 weeks. She visited the emergency department with sudden pain, numbness, and swelling in her left hand, 5 days after receiving the second cycle of chemotherapy.

## 4 Diagnostic focus and assessment

**Figure 2:**
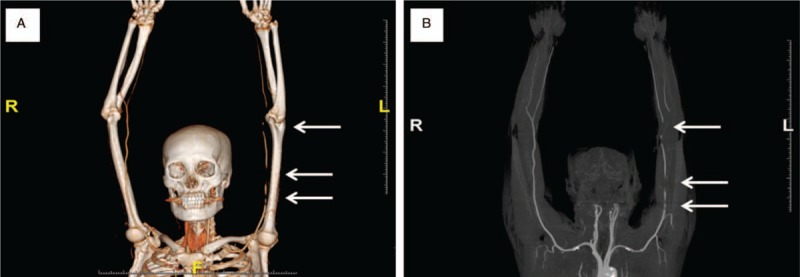
CT angiography 3-dimensional image findings showed multifocal total occlusion of left brachial artery (white arrows) with reconstitution at proximal radial and ulnar arteries suggesting acute thrombosis (A, B).

On physical examination, her left forearm and hand were cold, edematous, and painful, with a weak left radial artery pulse. The Allen test was positive and the doppler flow signal was not present in the left brachial and distal bifurcating arteries, suggestive of possible arterial occlusion. On the CT angiography study, a thrombus was noted in a segment of the brachial artery and at the junction of the brachial and radioulnar arteries (Fig. 2). Significant perivascular hazy edematous changes were suggestive of extensive acute occlusive arterial thrombosis. The laboratory findings related to coagulation were as follows: PT: 10.4 seconds (range: 0.1–12.1), PT/INR: 0.89 (<1.2), aPTT: 15.6 seconds (range: 24.0–33.0), fibrin degradation products (FDP): 8.7 ug/mL (range: <5), D-dimer: 3.8 ug/mL (range: <0.5), protein C: 65% (range: 60–150), protein S: 107% (range: 70%–130%), antiphospholipid antibody: 2.0 U/ mL (range <10), lupus anticoagulant: 0.87 (negative: <1.20), antithrombin III: 52.3% (range: 74–125), factor VIII ab (inhibitor): 90% (negative range: 81%–100%). Echocardiography demonstrated no intracardiac thrombi.

## 5 Therapeutic focus and assessment

**Figure 3:**
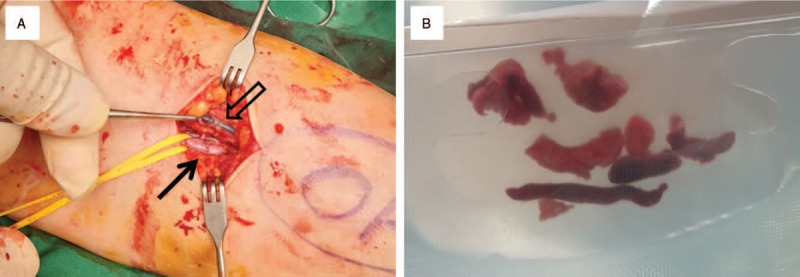
Operative field exposing left brachial artery and cephalic vein (A). Brachial artery (black arrow) was repaired after thrombectomy and cephalic vein (black empty arrow) was also filled by thrombus. Specimen after thrombectomy conclusive of acute arterial thrombosis (B).

The patient immediately received 10,000 units of unfractionated heparin (UFH). She then underwent emergency brachial and radial-ulnar thrombectomy under local anesthesia in operating room with restoration of perfusion to the extremity (Fig. 3). The postoperative course was uncomplicated, and her symptoms subsided completely after surgery. After continuous intravenous UFH followed by warfarin, and she discharged on warfarin at a daily dose of 4 mg after 5 days.

## 6 Follow-up and outcomes

Ten days after surgery, the PT/INR level was 1.25 (<1.2) with daily warfarin dose of 4 mg. After multidisciplinary team meeting, we decided to discontinue chemotherapy permanently and anticoagulation with warfarin was continued. The patient started treatment with adjuvant endocrine therapy with aromatase inhibitor (AI); anastrozole 1 mg daily. Adjuvant radiotherapy was administered to her right breast, consisting of 50Gy in 25 fractions. She continued taking the warfarin 4 months, with a target therapeutic PT/INR level of 1.5–2. She was asymptomatic at follow-up at four months, and, due to the complete resolution of symptoms, oral anticoagulation was discontinued. At her 10-month follow-up, she was asymptomatic and found to have no concerning thromboembolic issues. She is doing well, is currently on endocrine therapy with no evidence of breast cancer recurrence.

## 7 Discussion

Arterial thrombosis occurs in 1.0% to 4.8% of patients treated with multiagent chemotherapy for breast cancer.^[7,9,10,11]^ Most thrombotic complications occur during treatment rather than after completion of chemotherapy.^[9,11]^ The risk is greater in patients with metastatic disease, probably owing to the increased tumor burden and the presence of other predisposing factors, such as immobilization.^[11]^ The incidence of thrombosis is reported to be higher in postmenopausal women and after mastectomy. Among premenopausal patients, the combination of chemotherapy and tamoxifen is associated with arterial thrombosis. Hormonal receptor status, age, the number of involved lymph nodes, and subsequent tumor recurrence apparently have no relation with the development of thrombosis.^[10]^

The pathophysiologic mechanism for thrombogenicity in patients undergoing chemotherapy for breast cancer is not well understood. Some studies have reported a statistically significant decrease in protein C and protein S levels, and a decrease in levels of factor VII and fibrinogen without clinically evident thrombosis during CMF chemotherapy for breast cancer.^[12,13]^ Reduced levels of protein C activity and increased levels of fibrinopeptide A have also been reported with infusional 5-fluorouracil.^[14]^ Possible explanations of these abnormalities include impairment of vitamin K metabolism, inhibition of DNA/RNA synthesis leading to a decrease in protein synthesis by the liver, and inhibition of intravascular coagulation.

In patients with breast cancer, chemotherapy regimens such as CMF, FAC (fluorouracil, Adriamycin, and cyclophosphamide), and CMFVP are known to increase the risk of thrombosis. Cisplatin based chemotherapy is also commonly implicated in the promotion of arterial and venous thrombosis in patients with advanced breast cancers.^[15]^ Angiogenesis inhibitors such as bevacizumab may cause both, thrombotic and hemorrhagic complications. In addition, supportive therapeutic agents such as erythropoietin, high dose corticosteroids as antiemetics, and granulocyte colony-stimulating factor (G-CSF) may increase the risk of vascular thrombosis.^[4]^

A few studies have reported on the occurrence of vascular thrombosis in patients receiving doxorubicin based chemotherapy for breast cancer.^[8,16]^ Although 10% of the patients developed venous thrombosis in the prospective study by Tempelhoff et al, advanced coagulation tests including levels of D-dimer, fibrinogen, and plasminogen activator inhibitor-1 (PAI) activity during adjuvant EC (epirubicin-cyclophosphamide) chemotherapy for breast cancer did not identify patterns of higher risk for DVT.^[8]^ In another study, the levels of plasma TAF1 antigen and PAI-1 were similar before and after Adriamycin based adjuvant chemotherapy in patients with operable breast cancer.^[17]^

In view of these reports, our patient had a number of distinct features. First, except for well controlled hypertension, she had an ECOG performance status score of 1, no atherosclerotic disease, a BMI of 22.9 kg/m^2^, and good levels of activity. She had no predisposing factors that could compromise arterial blood flow. Corticosteroids were administered as antiemetics. However, the dose was not likely to cause thromboembolism.

Second, a very rare report described a case of arterial thrombosis associated with adjuvant Adriamycin based chemotherapy. We found only 1 report of lower extremity arterial thrombosis during adjuvant dose dense AC chemotherapy with prophylactic G-CSF in breast cancer after mastectomy.^[16]^ These 2 reports suggest the possibility of serious arterial thrombosis with Adriamycin based chemotherapy.

In this patient, the anti-thrombin III (AT III) activity was below the lower limit of normal (74%–125%). However, she did not have any past history of TEEs. Also, in a cohort with familial AT III deficiency, the lack of AT III had not been regarded as a risk factor for arterial thrombosis.^[18]^ In familial studies, venous thrombosis occurred in 85% of AT-deficient relatives younger than 55 years of age. A large patient series with natural anticoagulant deficiency, including AT deficiency, revealed no increased risk of arterial cardiovascular disease in affected family members aged older than 55.^[19]^ AT levels should not be measured at the time of an event because thrombosis may cause a transient reduction in all natural anticoagulants including AT; this could be mistaken for an underlying deficiency. If the level of AT is found to be low during acute thrombosis, the estimation should be repeated once the patient has recovered.^[20]^ Therefore, we speculate that the low AT III activity in this case was not related to the arterial thrombosis. The test for the AT III level should have been repeated after the patient had completely recovered.

According to the 8th American college of chest physicians (AACP) consensus conference of antithrombotic therapy,^[21]^ the treatment of arterial thrombosis should include immediate initiation of anticoagulation with UFH and immediate thrombolytic therapy or operative intervention. The initiation of heparin infusions should be prompt and should not await results of diagnostic tests or procedures. In patients undergoing embolectomy, guidelines recommend the sequential administration of UFH and long-term anticoagulation with vitamin K antagonists as a grade 2C recommendation. In patients with active DVT and pulmonary embolism (PE), extended duration low molecular weight heparin (LMWH) for at least 6 months is currently the standard of care.^[22]^ Long-term LMWH is preferred over warfarin in the treatment of cancer-associated VTE to prevent recurrence.^[23]^ Extended anticoagulation beyond the standard 6 months should be considered as the optimal duration of treatment, particularly in those with active cancer and/or receiving anticancer treatments.^[22,24]^

In this case, the patient immediately received 10,000 units of heparin followed by continuous heparin infusion. She also underwent immediate operative thrombectomy. The patient then received UFH followed by warfarin. Since she had undergone curative resection for stage I breast cancer with a relatively low risk of recurrence, further chemotherapy was discontinued and anticoagulation was continued with warfarin. She subsequently received adjuvant radiotherapy to the right breast and adjuvant endocrine therapy with an AI.

This patient could have been prescribed an AI instead of tamoxifen as she was postmenopausal. Tamoxifen is well-known for increasing the risk of thromboembolism.^[1,2]^ As evident from randomized clinical trials, thrombosis in women receiving endocrine adjuvant therapy for early breast cancer is substantially lower with AIs compared to tamoxifen.^[25,26]^ However, it is unclear whether AIs are completely unassociated with thrombotic risks. Longer follow up is therefore needed.

Arterial thrombosis complicating cancer and cancer chemotherapy are common, and has increased with the expansion of the armamentarium of novel chemotherapeutic agents. Owing to the improved survival in patients with cancer, cardiovascular complications are becoming clinically apparent many years after the diagnosis of cancer. Thromboprophylaxis should be considered in patients with cancer. Notably, the guidelines of the American Society of Clinical Oncology (ASCO) and National Comprehensive Cancer Network (NCCN) recommend thromboprophylaxis only for in-patients without contraindications for anticoagulation.^[27,28]^ These recommendations are based on results of large trials conducted in medically ill patients, of whom a minority of patients had cancer. Cancer-specific studies have not been conducted. Some randomized controlled trials studied the prophylactic use of LMWH or ultra-LMWH among outpatients with common cancers or single site cancers known to be at a very high risk for VTE (pancreas and multiple myeloma). Patients receiving prophylaxis showed a significant reduction in the relative risk of VTE (hazard ratio [HR]: 0.36).^[29–32]^ However, since VTE events were relatively low among the patients with common cancers in those studies (3.2% and 3.4%), risk stratification was necessary. Khorana et al. proposed a predictive model for chemotherapy- associated VTE based on the site of cancer (very high risk for stomach and pancreas), pre-chemotherapy platelet count (>350,000/mm^3^), hemoglobin level (<10 g/dL), leukocyte count (>11,000/mm^3^), and BMI (≥35 kg/m^2^). Patients were stratified as being at high, intermediate, and low risk for the development of VTE. In studies that validated risk scores, patients with high risk scores (≥3) had a 5.4% to 28.2% incidence of VTEs, while those with low risk scores had an incidence of 1.5% to 3%.^[24,33]^ The risk for chemotherapy-induced VTE may be predicted using the model proposed by Khorana et al; it may also be used to predict the risk for arterial thrombosis. However, the current ASCO and NCCN guidelines recommend outpatient thromboprophylaxis only for high-risk myeloma patients receiving thalidomide lenalidomide- or pomalidomide -based combination regimens.^[27,28]^ Evidence based changes in current treatment algorithms are urgently needed, with further studies on thromboprophylaxis for arterial thrombosis in cancer patients.

Cancer is associated with an increased incidence of thrombosis. However, the point of initiation of the increased risk remains unclear. Retrospective case control studies using the SEER dataset have shown that in patients ≥67 years old, the risk of arterial thromboembolic events begin to increase 150 days before the date of cancer diagnosis, with a peak at 30 days before.^[34]^ This finding suggests that a new diagnosis of myocardial infarction or ischemic stroke in older patients should provoke age-appropriate screening for malignancy. The findings also confirm the importance of risk group analysis and thromboprophylaxis in high-risk patients with cancer.

In conclusion, we report an extremely rare case of arterial thrombosis in a patient with early breast cancer receiving adjuvant chemotherapy with Adriamycin-cyclophosphamide after curative resection. Despite the relatively low incidence, arterial thrombosis is a potentially devastating complication with high mortality. Careful clinical observation is important in patients receiving chemotherapy, even when the primary tumor has been completely resected. Patients should be carefully chosen for prophylaxis. Randomized trials are necessary to identify patients who may benefit from thromboprophylaxis while on multiagent chemotherapy.

## Author contributions

**Data curation:** Lee Chan Jang, Young Jin Choi.

**Formal analysis:** Young Jin Choi.

**Investigation:** Lee Chan Jang, Young Jin Choi.

**Methodology:** Lee Chan Jang, Young Jin Choi.

**Resources:** Young Jin Choi.

**Writing – original draft:** Young Jin Choi.

**Writing – review & editing:** Lee Chan Jang, Young Jin Choi.

Young Jin Choi orcid: 0000-0003-1030-4211.

## References

[1] Caine GJ Stonelake PS Rea D Coagulopathic complications in breast cancer. Cancer 2003;98:1578–86. 14534872 10.1002/cncr.11702

[2] Levine MN Adjuvant therapy and thrombosis: how to avoid the problem? Breast 2007;16: suppl 2: S169–74. 17720502 10.1016/j.breast.2007.07.012

[3] Otten HM Mathijssen J ten Cate H Symptomatic venous thromboembolism in cancer patients treated with chemotherapy: an underestimated phenomenon. Arch Intern Med 2004;164:190–4. 14744843 10.1001/archinte.164.2.190

[4] Haddad TC Greeno EW Chemotherapy-induced thrombosis. Thromb Res 2006;118:555–68. 16388837 10.1016/j.thromres.2005.10.015

[5] Rella C Coviello M Giotta F A prothrombic state in breast cancer patients treated with adjuvant chemotherapy. Breast Cancer Res Treat 1996;40:151–9. 8879681 10.1007/BF01806210

[6] Goodnough LT Saito H Manni A Increased incidence of thromboembolism in Stage IV breast cancer patients treated with a five-drug chemotherapy regimen. A study of 159 patients. Cancer 1984;54:1264–8. 6547874 10.1002/1097-0142(19841001)54:7<1264::aid-cncr2820540706>3.0.co;2-r

[7] Weiss RB Tormey DC Holland JF Venous thrombosis during multimodal treatment of primary breast carcinoma. Cancer Treat Rep 1981;65:677–9. 7248984

[8] von Tempelhoff GF Dietrich M Hommel G Blood coagulation during adjuvant epirubicin/cyclophosphamide chemotherapy in patients with primary operable breast cancer. J Clin Oncol 1996;14:2560–8. 8823336 10.1200/JCO.1996.14.9.2560

[9] Levine MN Gent M Hirsh J The thrombogenic effect of anticancer drug therapy in women with stage II breast cancer. N Engl J Med 1988;318:404–7. 3340118 10.1056/NEJM198802183180703

[10] Saphner T Tormey DC Gray R Venous and arterial thrombosis in patients who received adjuvant therapy for breast cancer. J Clin Oncol 1991;9:286–94. 1988575 10.1200/JCO.1991.9.2.286

[11] Wall JG Weiss RB Norton L Arterial thrombosis associated with adjuvant chemotherapy for breast carcinoma: a Cancer and Leukemia Group B Study. Am J Med 1989;87:501–4. 2510514 10.1016/s0002-9343(89)80604-7

[12] Rogers JS Murgo II Fontana AJ PC Chemotherapy for breast cancer decreases plasma protein C and protein S. J Clin Oncol 1988;6:276–81. 2963094 10.1200/JCO.1988.6.2.276

[13] Feffer SE Carmosino LS Fox RL Acquired protein C deficiency in patients with breast cancer receiving cyclophosphamide, methotrexate, and 5-fluorouracil. Cancer 1989;63:1303–7. 2920359 10.1002/1097-0142(19890401)63:7<1303::aid-cncr2820630713>3.0.co;2-f

[14] Kuzel T Esparaz B Green D Thrombogenicity of intravenous 5-fluorouracil alone or in combination with cisplatin. Cancer 1990;65:885–9. 2297659 10.1002/1097-0142(19900215)65:4<885::aid-cncr2820650410>3.0.co;2-h

[15] Centola M Lucreziotti S Cazzaniga S A rare case of large intracoronary thrombosis in advanced breast cancer patient treated with epirubicin and cisplatin. J Cardiovasc Med (Hagerstown) 2016;17: Suppl 2: e241–3. 27606783 10.2459/JCM.0000000000000444

[16] Bourdeanu L Luu T Arterial thrombosis associated with adjuvant chemotherapy for breast cancer: a case report. J Am Acad Nurse Pract 2010;22:140–3. 20236397 10.1111/j.1745-7599.2009.00486.x

[17] Demirkan B Ozcan MA Glu AA The efffect of anthracycline-based (epirubicin) adjuvant chemotherapy on plasma TAF1 and PAI-1 levels in operable breast cancer. Clin Appl Thromb Hemost 2006;12:9–14. 16444429 10.1177/107602960601200103

[18] Mahmoodi BK Brouwer JL Veeger NJ Hereditary deficiency of protein C or protein S confers increased risk of arterial thromboembolic events at a young age: results from a large family cohort study. Circulation 2008;118:1659–67. 18824642 10.1161/CIRCULATIONAHA.108.780759

[19] Boekholdt SM Kramer MH Arterial thrombosis and the role of thrombophilia. Semin Thromb Hemost 2007;33:588–96. 17768691 10.1055/s-2007-985755

[20] Patnaik MM Moll S Inherited antithrombin deficiency: a review. Haemophilia 2008;14:1229–39. 19141163 10.1111/j.1365-2516.2008.01830.x

[21] Sobel M Verhaeghe R Antithrombotic therapy for peripheral artery occlusive disease: American College of Chest Physicians Evidence-Based Clinical Practice Guidelines (8th Edition). Chest 2008;133: suppl 6: 815S–43S. 18574279 10.1378/chest.08-0686

[22] Brose KM Lee AY Cancer-associated thrombosis: prevention and treatment. Curr Oncol 2008;15: suppl 1: S58–67. 18231650 10.3747/co.2008.177 PMC2216419

[23] Lee AY Levine MN Baker RI Low-molecular-weight heparin versus a coumarin for the prevention of recurrent venous thromboembolism in patients with cancer. N Engl J Med 2003;349:146–53. 12853587 10.1056/NEJMoa025313

[24] Khorana AA Cancer-associated thrombosis: updates and controversies. Hematol Am Soc Hematol Educ Program 2012;2012:626–30. 10.1182/asheducation-2012.1.626 23233644

[25] Baum M Budzar AU Cuzick J Anastrozole alone or in combination with tamoxifen versus tamoxifen alone for adjuvant treatment of postmenopausal women with early breast cancer: first results of the ATAC randomised trial. Lancet 2002;359:2131–9. 12090977 10.1016/s0140-6736(02)09088-8

[26] Coates AS Keshaviah A Thürlimann B Five years of letrozole compared with tamoxifen as initial adjuvant therapy for postmenopausal women with endocrine-responsive early breast cancer: update of study BIG 1-98. J Clin Oncol 2007;25:486–92. 17200148 10.1200/JCO.2006.08.8617

[27] National Comprehensive Cancer Network Clinical Practice Guidelines in Oncology, Cancer-Associated Venous Thromboembolic Disease (V.1.2019) Accessed February 28, 2019.

[28] Lyman GH Bohlke K Khorana AA Venous thromboembolism prophylaxis and treatment in patients with cancer: American society of clinical oncology clinical practice guideline update 2014. J Clin Oncol 2015;33:654–6. 25605844 10.1200/JCO.2014.59.7351 PMC4881372

[29] Agnelli G Gussoni G Bianchini C Nadroparin for the prevention of thromboembolic events in ambulatory patients with metastatic or locally advanced solid cancer receiving chemotherapy: a randomised, placebo-controlled, double blind study. Lancet Oncol 2009;10:943–9. 19726226 10.1016/S1470-2045(09)70232-3

[30] Agnelli G George DJ Kakkar AK Semuloparin for thromboprophylaxis in patients receiving chemotherapy for cancer. N Engl J Med 2012;366:601–9. 22335737 10.1056/NEJMoa1108898

[31] Pelzer U Opitz B Deutschinoff G Efficacy of prophylactic low-molecular weight heparin for ambulatory patients with advanced pancreatic cancer: outcomes from the CONKO-004 trial. J Clin Oncol 2015;33:2028–34. 25987694 10.1200/JCO.2014.55.1481

[32] Palumbo A Cavo M Bringhen S Aspirin, warfarin, or enoxaparin thromboprophylaxis in patients with multiple myeloma treated with thalidomide: a phase III, open-label, randomized trial. J Clin Oncol 2011;29:986–93. 21282540 10.1200/JCO.2010.31.6844

[33] Khorana AA Kuderer NM Culakova E Development and validation of a predictive model for chemotherapy-associated thrombosis. Blood 2008;111:4902–7. 18216292 10.1182/blood-2007-10-116327 PMC2384124

[34] Navi BB Reiner AS Kamel H Arterial thromboembolic events preceding the diagnosis of cancer in older persons. Blood 2019;133:781–9. 30578253 10.1182/blood-2018-06-860874 PMC6384185

